# Genome Sequence of Staphylococcus aureus Strain PS/BAC/317/16/W, Isolated from Contaminated Platelet Concentrates in England

**DOI:** 10.1128/MRA.00577-21

**Published:** 2021-09-02

**Authors:** Basit Yousuf, Annika Flint, Kelly Weedmark, Carl McDonald, Jennifer Bearne, Franco Pagotto, Sandra Ramirez-Arcos

**Affiliations:** a Centre for Innovation, Canadian Blood Services, Ottawa, Canada; b Department of Biochemistry, Microbiology and Immunology, University of Ottawa, Ottawa, Canada; c Bureau of Microbial Hazards, Health Products and Food Branch, Health Canada, Ottawa, Canada; d National Health Service Blood and Transplant, London, United Kingdom; University of Maryland School of Medicine

## Abstract

We present the genome sequence of Staphylococcus aureus strain PS/BAC/317/16/W, which was isolated from contaminated platelet concentrates by the National Health Service Blood and Transplant in England (2017). Genome sequence analysis revealed the presence of one chromosome (2,665,983 bp) and two plasmids (4,265 bp and 2,921 bp) in this strain.

## ANNOUNCEMENT

Staphylococcus aureus, an opportunistic pathogen, is part of the normal mucosa flora, which can colonize human skin (1). S. aureus is occasionally introduced into platelet concentrates (PCs) during venipuncture at the time of blood collection. The pretransfusion storage requirements of PCs at room temperature provide a favorable environment for S. aureus proliferation. In some countries, PCs are routinely screened for bacterial contamination using automated culture methods before being released for transfusion (2). However, S. aureus is occasionally missed during PC screening, resulting in post-transfusion septic reactions (2–4). In this report, we share the genome sequence of S. aureus PS/BAC/317/16/W, which was undertaken as part of a hemovigilance investigation of bacterial contamination in PCs.

S. aureus PS/BAC/317/16/W was isolated from contaminated PCs during screening using the BACT/ALERT culture system following standard procedures at the National Health Service Blood and Transplant (NHSBT). After S. aureus identification, the isolate was stored frozen at –80°C. For DNA isolation, S. aureus PS/BAC/317/16/W was streaked onto blood agar plates, and single colonies were cultured at 35°C overnight in 5 ml Trypticase soy broth with 0.6% yeast extract (5). Cells were collected by centrifugation and resuspended in DNA/RNA Shield reagent. DNA extraction was performed using the Zymo Quick-DNA high-molecular-weight (HMW) MagBead kit (Cedarlane) with lysozyme and RNase A treatment as per the manufacturer’s instructions (Zymo Research Corp.).

Illumina paired-end (2 × 300-bp) whole-genome shotgun (WGS) sequence data were generated using Nextera XT DNA libraries run on a MiSeq instrument (v3 chemistry) according to the manufacturer’s instructions (Illumina, Inc.). The Illumina reads (*n* = 2,034,308) were processed using fastp v0.20.0 (6) to remove adapter and barcode sequences, correct mismatched bases in overlaps, and filter low-quality reads, resulting in 1,813,396 filtered reads. Oxford Nanopore data (199,747 reads) were obtained using the rapid barcoding sequencing kit (SQK-RBK004) and 1D MinION chemistry (R9.4 FLO-MIN106 flow cell) as per the manufacturer’s protocol (Oxford Nanopore Technologies). Long-read signal processing, base calling, demultiplexing, and adapter trimming were performed using Guppy GPU v3.3.3+fa743ab, and reads of <1 kb were removed using Filtlong v0.2.0 (https://github.com/rrwick/Filtlong), resulting in 163,856 filtered reads (*N*_50_, 8,010 bp).

A *de novo* hybrid assembly using the filtered MinION and Illumina reads was generated with Trycycler v0.3.3 (cluster, reconcile, partition, and consensus functions; default circularization and rotation), Flye v2.8.1-b1676 Minipolish+Miniasm v0.1.2, Wtdbg2 v2.5, and Raven v1.2.2 with error correction (Medaka v1.1.3 and Pilon v1.23) (7). For all computational tools, default parameters were used except where otherwise noted.

The complete, closed S. aureus PS/BAC/317/16/W genome sequence contains a 2,665,983-bp chromosome and two plasmids (4,265 bp and 2,921 bp) and has an average coverage depth of 243-fold for the Illumina data set and 333-fold for the Nanopore data set. This genome has an average GC content of 32.92% and 2,592 annotated features, including 2,393 genes, 118 pseudogenes, 0 CRISPRs, 19 rRNAs, 58 tRNAs, and 4 noncoding RNAs (ncRNAs).

PS/BAC/317/16/W was checked for multilocus sequence typing (MLST) using the PubMLST database (8); however, no sequence type (ST) was assigned to this genome.

### Data availability.

This whole-genome shotgun project is available in GenBank and the Sequence Read Archive under the accession numbers in Table 1.

**TABLE 1:** Provenance and accession numbers linked to the NCBI database for isolate PS/BAC/317/16/W

Parameter	Data
Isolate	PS/BAC/317/16/W
Bioproject accession no.	PRJNA703976
GenBank accession no.	CP071104, CP071105, CP071106
Illumina and Nanopore SRA accession no.	SRR13745242, SRR13745248
Country (region)	UK (England)
Yr	2016
